# Supplementary material for: Guard cells control hypocotyl elongation through HXK1, HY5, and PIF4
Source: Commun Biol. 2021 Jun 21;4:765. doi: 10.1038/s42003-021-02283-y (PMC8217561; doi:10.1038/s42003-021-02283-y)
Supplement: Supplementary file 1 — Supplementary Material [file 42003_2021_2283_MOESM1_ESM.pdf]

Guard cells control hypocotyl elongation through HXK, HY5 and PIF4

Gilor Kelly[1], Danja Brandsma[1], Aiman Egbaria[1,2], Ofer Stein[1], Adi Doron-Faigenboim[1], Nitsan Lugassi[1], Eduard Belausov[1], Hanita Zemach[1], Felix Shaya[1], Nir Carmi[1], Nir Sade[2] and David Granot[1*]

[1]*Institute of Plant Sciences, Agricultural Research Organization, The Volcani Center, Rishon LeZion 7505101, Israel*

[2]*School of Plant Science and Food Security, Tel Aviv University, Tel Aviv, Israel*

***Corresponding author:** David Granot, granot@agri.gov.il

This file includes:

Supplementary Tables 1-6

Supplementary Figures 1-6

Supplementary Material

Supplementary Table 1 qPCR primers used in this study.

Supplementary Table 2 Primers used for mutant genotyping.

Supplementary Table 3 Primers used for cloning and the identification of transgenic plants.

Supplementary Table 4 Accession numbers and probe ID numbers of *AtHY5* and *AtHYH* used in the microarray database analysis.

Supplementary Table 5 Optimized LC-MS-MS parameters for quantification of plant hormones.

Supplementary Table 6 Solvent gradient program for auxins

Supplementary Figure 1 Genes differentially expressed in GCHXK transgenic seedlings relative to the WT

Supplementary Figure 2 Pathways changed by GCHXK

Supplementary Figure 3 Gene ontology (GO) analysis of differentially expressed, down-regulated (a) biological processes, (b) cellular components and (c) molecular functions in GCHXK, relative to the WT

Supplementary Figure 4 Gene ontology (GO) analysis of differentially expressed, up-regulated (a) biological processes, (b) cellular components and (c) molecular functions in GCHXK, relative to the WT

Supplementary Figure 5 Distribution of GCHY5-GFP and chlorophyll autofluorescence in the epidermis of young seedlings

Supplementary Figure 6 HY5 and HYH expression in response to glucose

Supplementary Table 1. qPCR Primers used in this study

Gene	Primer sequence	Accession No.
**TUB2 f (a)**	AAACTCACTACCCCCAGCTTTG	At5g62690
**TUB2 r**	CACCAGACATAGTAGCAGAAATCAAGT	
**HXK1 f (a)**	GCCTTTGAAGAGGATTGTGC	At4g29130
**HXK1 r**	CATGACACGGAAGTTTGTCC	
**HY5 f**	TCAGAACGAGAACCAGATGC	AT5G11260
**HY5 r**	GAAGGAGATCAAAGGCTTGC	
**HY5 f**	TCAGAACGAGAACCAGATGCT	AT5G11260
**HY5 r**	CAGCATTAGAACCACCACCA	
**PIF1 f**	GGAGCTTTTATGGCAGAACG	AT2G20180
**PIF1 r**	AAATCATCGTCACGGAGAGG	
**PIF3 f**	ACGGACCACAGTTCCAAGTC	AT1G09530
**PIF3 r**	ATTGCCAATCGTCGAAGAAC	
**PIF4 f**	TCCTCAAGTCATGCCTCCTC	AT2G43010
**PIF4 r**	TAGGTCCAACGGTCGTTACC	
**PIF5 f**	TCAAAACCCGGTACAGTTGC	AT3G59060
**PIF5 r**	ACGGTCTGCATCTGATTTCC	
**SAUR50 f (b)**	CACTTCCCTGTCTATGTCGGA	AT4G34760
**SAUR50 r**	TCTTCCTCGGCTCGTTGTAA	
**SAUR65 f (b)**	AACAAAGAGCTGCCCTCAAGA	AT1G29460
**SAUR65 r**	AAACAGCCCTTCTCCACAGC	
**AtEXP8 f (c)**	CTCTTTCCGAAGAGTACCATGT	AT2G40610
**AtEXP8 r**	GTGTACGTCTCCTGCTCCTC	
**YUC8 f (c)**	CGATGAGACCAGTGGCTTGT	AT4G28720
**YUC8 r**	TTTTCTCCCGTAGCCACCAC	
**XTR7 f (c)**	CGGCTTGCACAGCCTCTT	AT4G14130
**XTR7 r**	TCGGTTGCCACTTGCAATT	
**PHYB f**	ACCGTCGTCAAGTCACACTC	AT2G18790
**PHYB r**	TGATTCGCCGGATTGTTCGA	
**COP1 f**	GGTACTCGCTGCGTGATTC	AT2G32950
**COP1 r**	GAGGAGTGTTCCAAGGCAAG	
**DET1 f**	TTCCACAACCACCCTACCTC	AT4G10180
**DET1 r**	GTAGCGAAAAGACCGAAACG	

(a) Primers adapted from Kelly *et al.* (2012)

(b) Primers adapted from Sun *et al.* (2016)

(c) Primers adapted from Gangappa *et al.* (2017)

Supplementary Table 2. Primers used for mutant genotyping

Gene	Accession No.	Primer sequence	Method
pif4-101 **(d)**	AT2G43010	LP-AATACATTTTGCAGGCAATCG<br>RP-CGTAATGAAGTTGCACGTTTACTC<br>LB3-TAGCATCTGAATTTCATAACCAATCTCGATACAC	PCR
**Cop1-4 (d)**	AT2G32950	GCCACATGAGAAGAACCAGATTG<br>TGAGTAGACAAGGAGGAACAAACC	Sequencing
hy5	AT5G11260	LP-ATTCCTTCCCAAAATGTCTCG<br>RP-ATGCGAGTGAATGACCATTTC<br>LBal TGGTTCACGTAGTGGGCCATCG	PCR

(d) Primers adapted from Pacin *et al.* (2016)

Supplementary Table 3. Primers used for cloning and transgenic plants identification

Promoter/gene	Primer sequence
**GCHXK**	TCTCAACAAATTCCCCTTGC<br>CATGACACGGAAGTTTGTCC
**GCHXK2 hemi/homo zygoute**	ACCAACCACCAACTAACCTCA<br>GCTCCCTGGATACAACGACA<br>TGCCTGCTTGCCGAATATCA
**35SHXK**	CAACCACGTCTTCAAAGCAA<br>CATGACACGGAAGTTTGTCC
**35SFRK1**	CAACCACGTCTTCAAAGCAA<br>AACATACGGCCGAACTCATC
**35SFRK2**	CAACCACGTCTTCAAAGCAA<br>ACGATGTTTCTATGCTCCTCCCT
**GCHY5**	TCTCAACAAATTCCCCTTGC<br>ACCACCTCCTCTCTTGTTTCC
**35SHY5**	CAACCACGTCTTCAAAGCAA<br>ACCACCTCCTCTCTTGTTTCC
**GCPIF4**	TCTCAACAAATTCCCCTTGC<br>ACCGGGATTGTTCTGAATTG
GCHY5-GFP	TCTCAACAAATTCCCCTTGC<br>ACCACCTCCTCTCTTGTTTCC<br>ACTGGGTGCTCAGGTAGTGG

Supplementary Table 4. Accession numbers and probe ID numbers of *AtHY5*, *AtHYH* used in the microarray database analysis

Gene name	Accession	Probe set ID
AtHY5	AT5G11260	250420_AT
AtHYH	AT3G1769	258349_AT

Supplementary Table 5. Optimized LC-MS-MS parameters for quantification of plants hormone.

Analite and IS	Retention time (min)	Ionization mode	MRM transition (m/z)	Dwell time (msec)	Cone (V)	Collision (V)
IAA	7.50	+	176>130<br>176>103	70	18	26<br>28
D5-IAA	7.42	+	181>134<br>181>106	70	20	16<br>30
IAAsp	5.50	+	291>134<br>291>130	100	20	12<br>20
DN-IAAsp	5.40	+	297>136<br>297>134	100	20	12<br>22
IAGlu	5.77	+	305>148<br>305>130	100	22	12<br>16
DN-IAGlu	5.81	+	311>150<br>311>134	100	20	12<br>20
OxIAA	5.18	+	192>146<br>192>128	50	14	14<br>24

Supplementary Table 6. Solvent gradient program for auxins

Time (min)	Phase A %	Phase B %
**Initial**	95	5
**0.5**	95	5
**14**	50	50
**15**	5	95
**18**	5	95
**19**	95	5
**22**	95	5

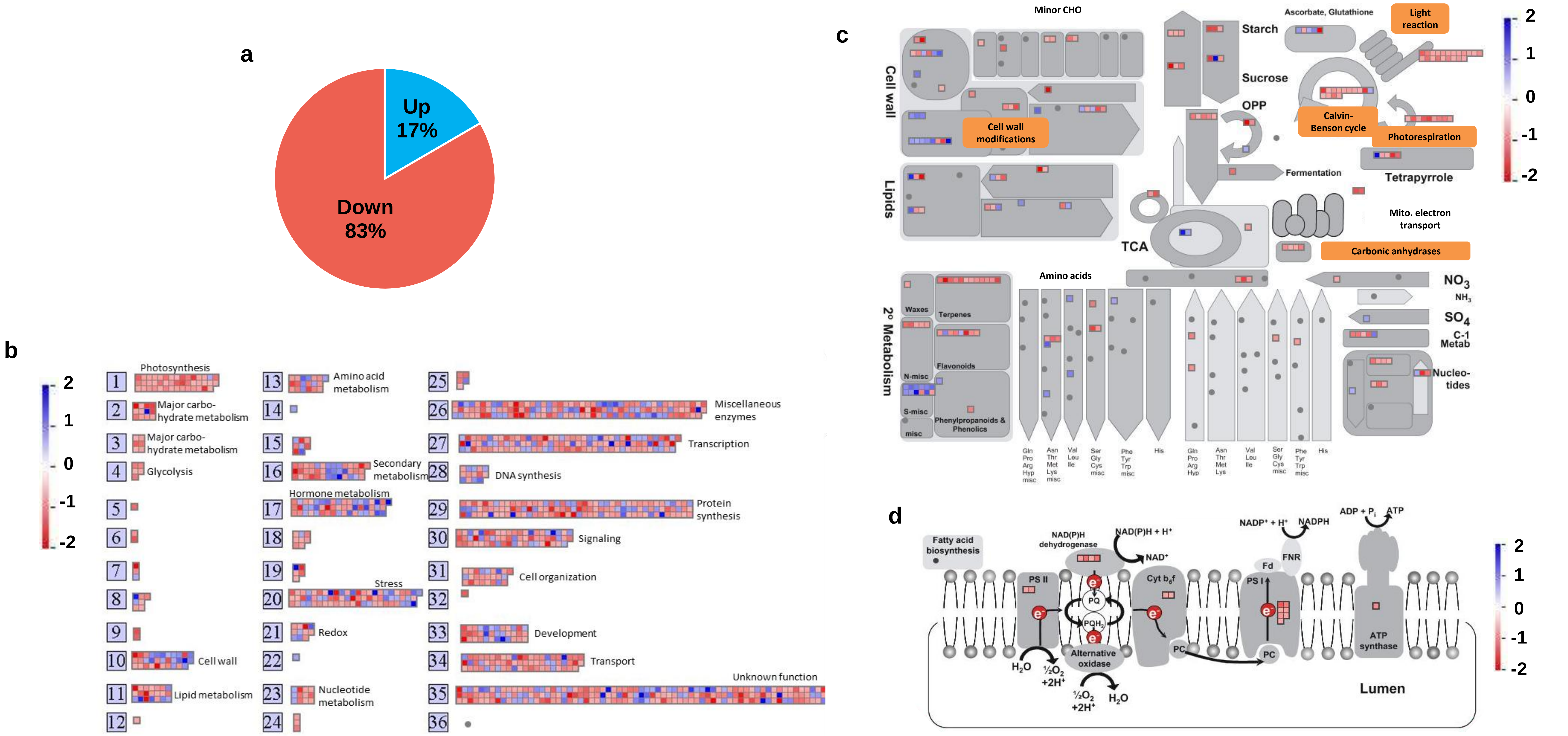

Supplementary Figure 1 Genes differentially expressed in GCHXK transgenic seedlings relative to the WT. Transcriptome analysis of 4-day-old GCHXK and WT seedlings. **a** The total number of transcripts that were up-regulated (blue) and down-regulated (red) in GCHXK relative to WT. **b** MapMan functional overview of transcript level changes in GCHXK seedlings relative to the WT. Genes are grouped into functional categories. **c** MapMan metabolism overview showing transcript level changes in GCHXK seedlings relative to WT. Light reactions, Calvin cycle, carbonic anhydrases and cell-wall groups are highlighted in orange. **d** GCHXK-regulated genes in the photosynthetic light reaction, as visualized by MapMan. **b-d** Genes up-regulated by GCHXK are stained blue and genes down-regulated by GCHXK are stained red.

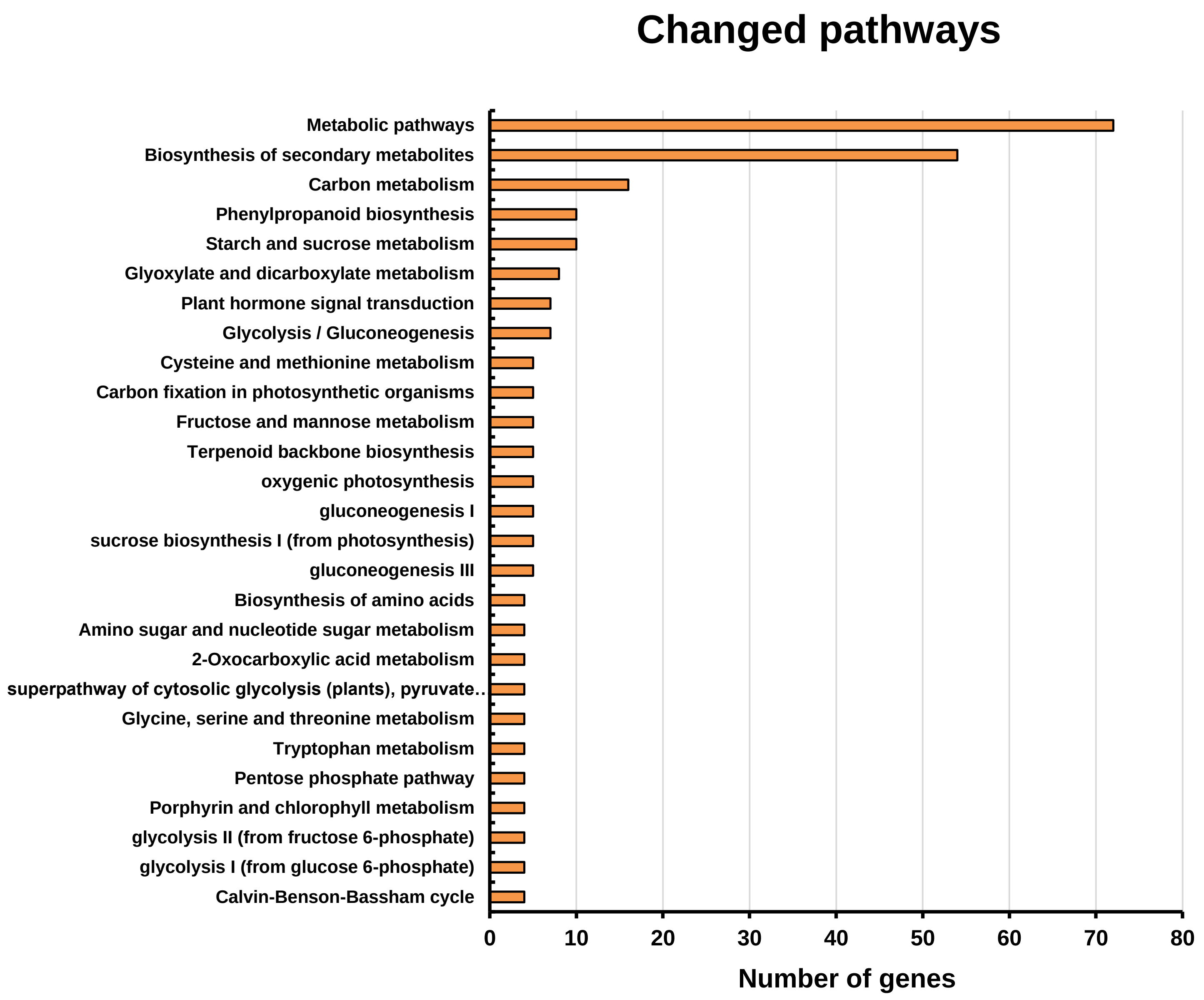

Supplementary Figure 2 Pathways changed by GCHXK. Pathways changed in GCHXK, relative to the WT, displayed by the number of genes significantly changed for each pathway. Changed pathways were analyzed by enrichment analysis using KOBAS (based on the KEGG PATHWAY and BioCyc databases). Pathways with more than three changed genes are displayed.

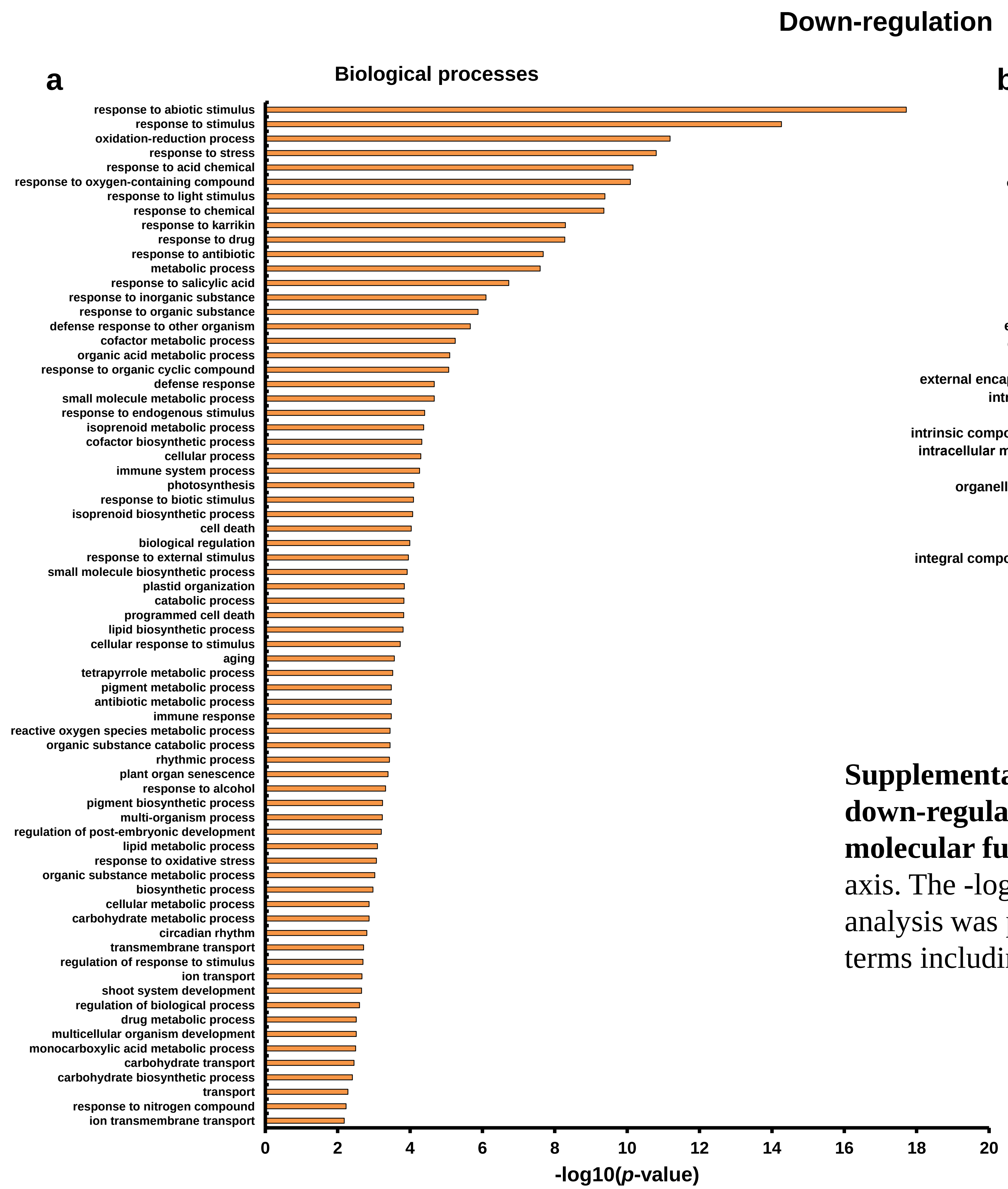

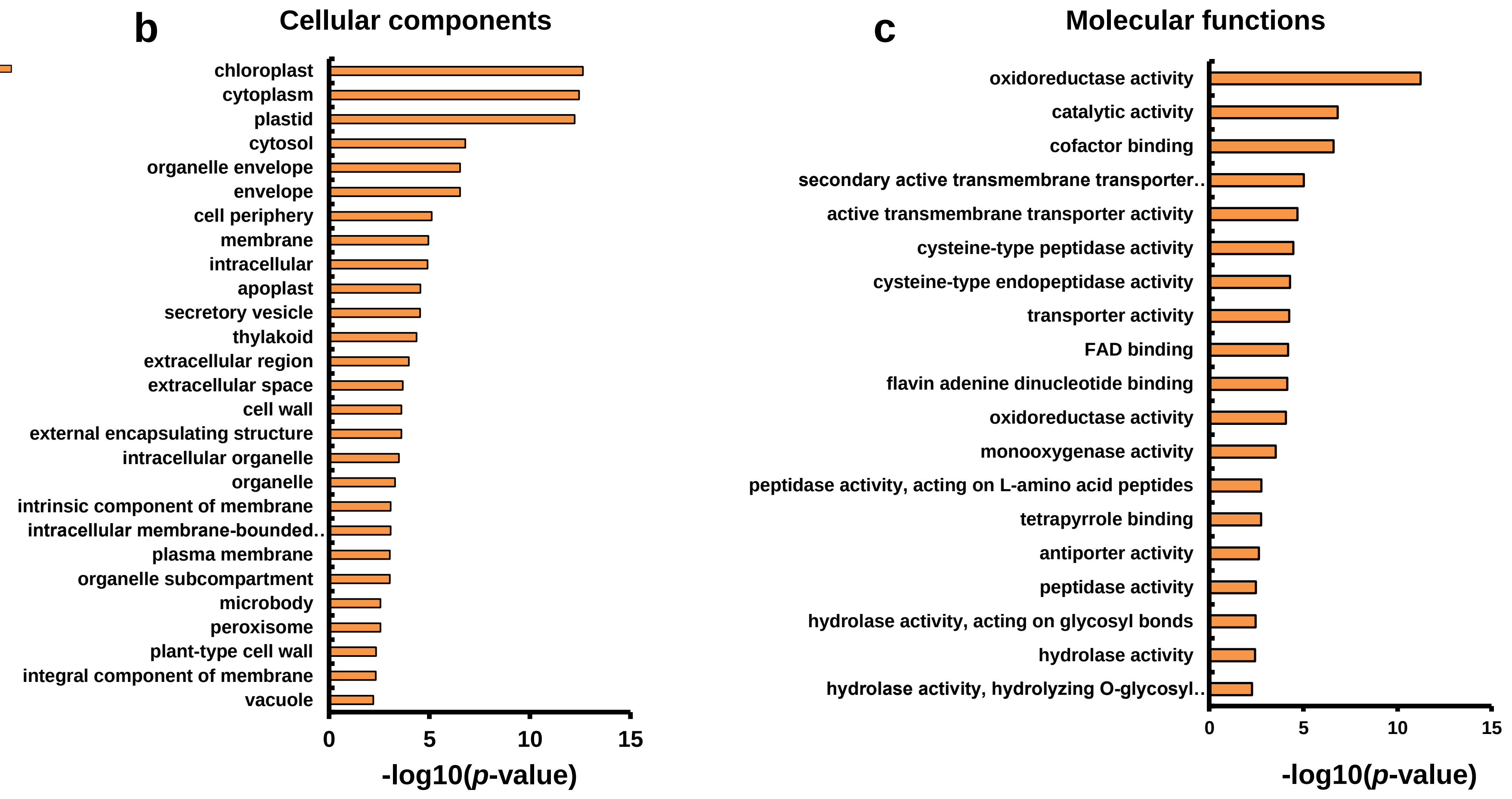

Supplementary Figure 3 Gene ontology (GO) analysis of differentially expressed, down-regulated (a) biological processes, (b) cellular components and (c) molecular functions in GCHXK, relative to the WT. GO terms are shown on the y-axis. The -log10 of the *P*-values of the GO terms are displayed on the *x*-axis. REVIGO analysis was performed on enriched GO terms with corrected *P*-values < 0.05. GO terms including at least five differently expressed genes are displayed.

Up-regulation

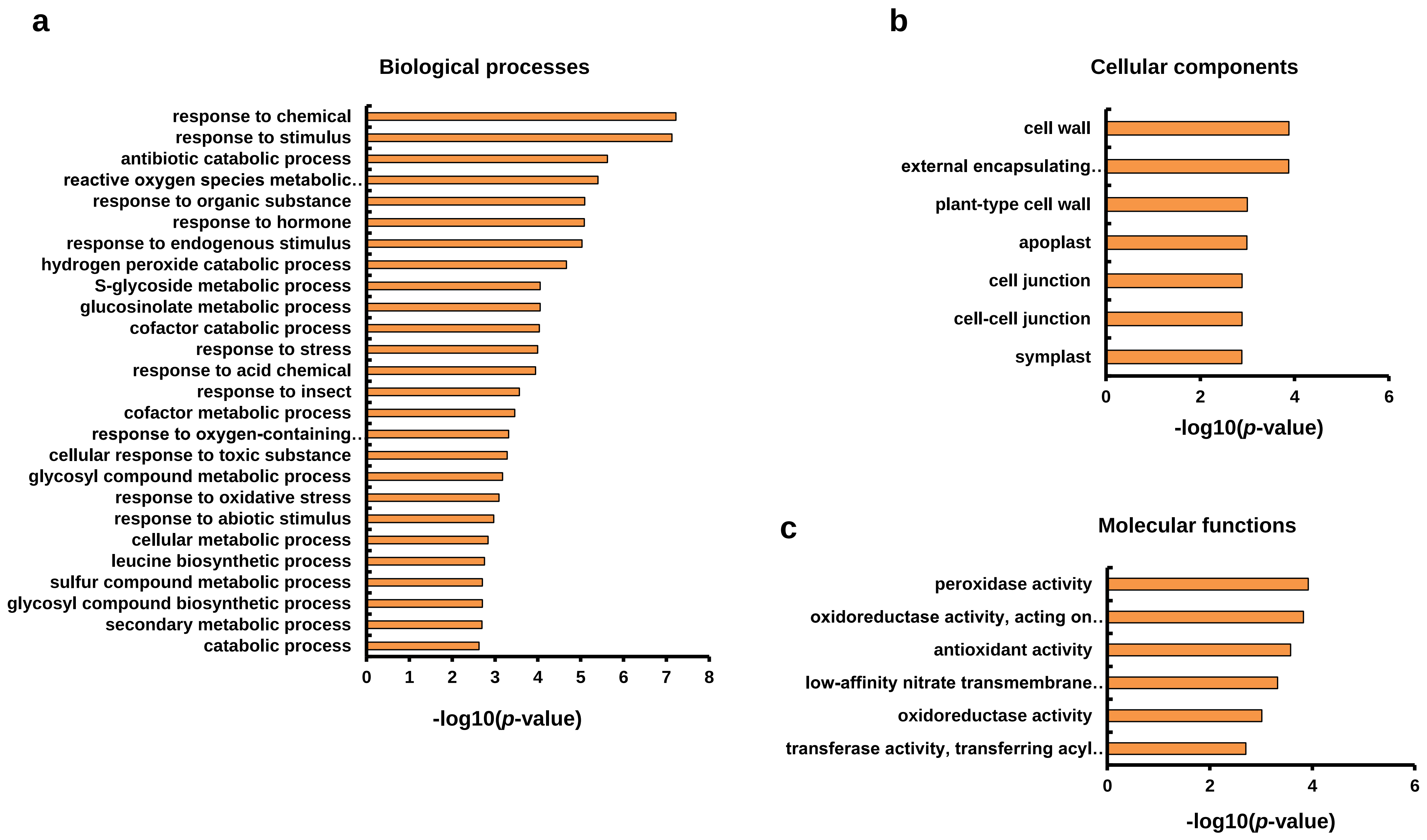

Supplementary Figure 4 Gene ontology (GO) analysis of differentially expressed, up-regulated (a) biological processes, (b) cellular components and (c) molecular functions in GCHXK, relative to the WT. GO terms are shown on the y-axis. The -log10 of the *P*-values of the GO terms are displayed on the x-axis. REVIGO analysis was performed on enriched GO terms with corrected *P*-values < 0.05.

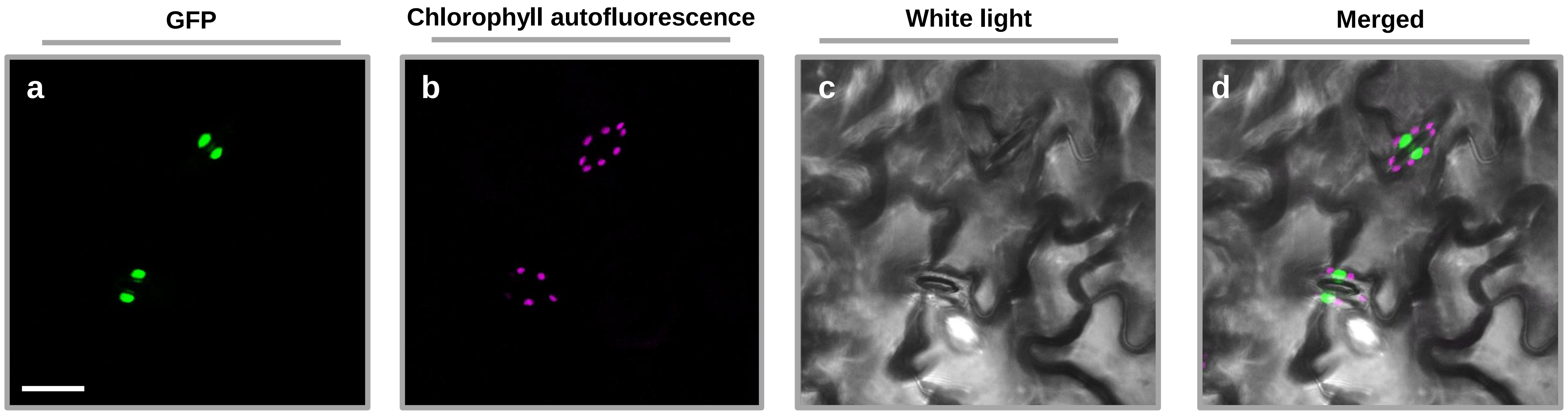

Supplementary Figure 5 Distribution of GCHY5-GFP and chlorophyll autofluorescence in the epidermis of young seedlings. Representative confocal images of GCHY5-GFP epidermis taken from the cotyledon of a 7-day-old seedling. **a** GFP fluorescence (stained green). **b** Chlorophyll autofluorescence (stained magenta). **c** White light. **d** Merged image of **(a-c)**. Bar = 20 μM.

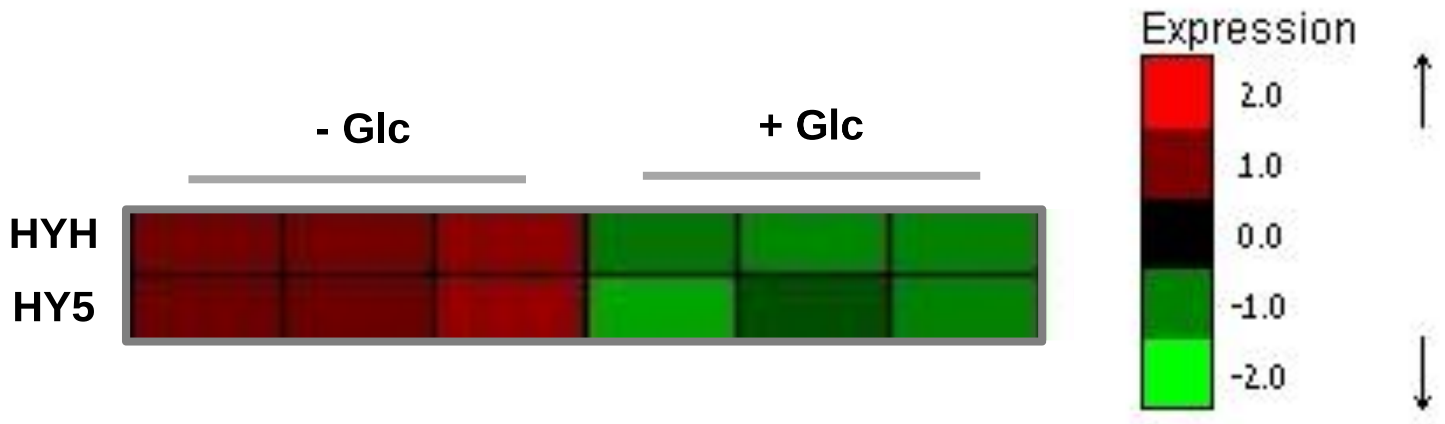

Supplementary Figure 6 HY5 and HYH expression in response to glucose. Data were obtained from the NASCArrays microarray database, Experiment No. 593, in which 7-day-old seedlings were exposed to 3% glucose for 6 h, followed by microarray analysis. The colors red and green represent induced or reduced expression, respectively, and the intensity of the color indicates the strength of expression. The accession number and the Affimetrix probe ID number for each gene are listed in Table S4.
