# Supplementary material for: Guard cells control hypocotyl elongation through HXK1, HY5, and PIF4
Source: Commun Biol. 2021 Jun 21;4:765. doi: 10.1038/s42003-021-02283-y (PMC8217561; doi:10.1038/s42003-021-02283-y)
Supplement: Supplementary file 3 — Description of Additional Supplementary Files [file 42003_2021_2283_MOESM3_ESM.pdf]

Description of Additional Supplementary Files

File Name: Supplementary Data 1
Description: Transcriptome analysis of 4-day-old WT and GCHXK seedlings.

File Name: Supplementary Data 2
Description: Gene ontology analysis of differentially expressed biological processes, cellular components and molecular functions in GCHXK, relative to the WT.
